# Liver-related safety assessment of green tea extracts in humans: a systematic review of randomized controlled trials

**DOI:** 10.1038/ejcn.2016.78

**Published:** 2016-05-18

**Authors:** T Isomura, S Suzuki, H Origasa, A Hosono, M Suzuki, T Sawada, S Terao, Y Muto, T Koga

**Affiliations:** 1 Clinical Study Support Inc., Nagoya, Japan; 2 Institute of Medical Science, Tokyo Medical University, Tokyo, Japan; 3 Department of Public Health, Nagoya City University Graduate School of Medical Sciences, Nagoya, Japan; 4 Division of Biostatistics and Clinical Epidemiology, University of Toyama Graduate School of Medicine and Pharmaceutical Sciences, Toyama, Japan

## Abstract

There remain liver-related safety concerns, regarding potential hepatotoxicity in humans, induced by green tea intake, despite being supposedly beneficial. Although many randomized controlled trials (RCTs) of green tea extracts have been reported in the literature, the systematic reviews published to date were only based on subjective assessment of case reports. To more objectively examine the liver-related safety of green tea intake, we conducted a systematic review of published RCTs. A systematic literature search was conducted using three databases (PubMed, EMBASE and Cochrane Central Register of Controlled Trials) in December 2013 to identify RCTs of green tea extracts. Data on liver-related adverse events, including laboratory test abnormalities, were abstracted from the identified articles. Methodological quality of RCTs was assessed. After excluding duplicates, 561 titles and abstracts and 119 full-text articles were screened, and finally 34 trials were identified. Of these, liver-related adverse events were reported in four trials; these adverse events involved seven subjects (eight events) in the green tea intervention group and one subject (one event) in the control group. The summary odds ratio, estimated using a meta-analysis method for sparse event data, for intervention compared with placebo was 2.1 (95% confidence interval: 0.5–9.8). The few events reported in both groups were elevations of liver enzymes. Most were mild, and no serious liver-related adverse events were reported. Results of this review, although not conclusive, suggest that liver-related adverse events after intake of green tea extracts are expected to be rare.

## Introduction

Green tea is widely consumed in Asia, especially in Japan and China.^1^ It has been a popular drink for a long time and is consumed by many people on a daily basis. Green tea contains mainly catechin, theanine and caffeine. Catechin components include epicatechin, epigallocatechin (EGC), epicatechin gallate and epigallocatechin gallate (EGCG), and about half of the total catechin is EGCG.^2^ Catechin is known as an antioxidant that prevents genetic damage caused by free radicals and is expected to protect against various types of cancer.

Besides a beverage, green tea can be consumed in a supplement form and is among the most commonly used dietary supplements in the United States.^3^ Green tea supplements are often taken for weight loss because of catechin's expected fat-burning effect. Unlike pharmaceuticals, supplements do not require regulatory approvals for dosage and administration, which may frequently result in consumption in excess of recommended amounts. Unfortunately, consecutive cases of liver damage seemingly caused by excessive consumption of green tea supplements have been reported, and this has become a safety concern. As a result, the green tea extract Exolise was withdrawn from the market in France and Spain in 2003.^4^

Several experiments using animals have shown acute hepatotoxicity of high-dose intakes of some green tea extracts; therefore, safety concerns have been raised regarding the possibility of chronic toxicity and, in particular, liver carcinogenicity.^5, 6^ Recently, the National Toxicology Program, part of the US Department of Health and Human Services, released a draft report on animal safety experiments in which extremely high doses of green tea extracts, much higher than those usually recommended for humans, were repeatedly administered.^7^ In 2-year gavage studies, liver toxicity (for example, hepatic necrosis) was frequently observed; however, there was no evidence of liver carcinogenicity of green tea extract. Similarly, other animal experiments showed no evidence of liver cancer when high concentrations of green tea extracts were tested.^8^ However, the results of animal experiments cannot be simply extrapolated to humans.

In order to investigate the hepatic safety of green tea extracts in humans, Sarma *et al.*^3^ conducted a systematic review of case reports published from 1966 to 2007. Among the 216 cases that were extracted, 34 were related to liver damage, with a causal relationship to green tea extract possible in 27 cases and probable in 7. In another review of case reports published from 1999 to 2008, including two unpublished reports,^9^ a total of 36 cases of liver damage were reported, including 13 cases of duplication with Sarma *et al.*^3^ This review concluded that, although a causal relationship to green tea extracts was suggested, the effects of concomitant drugs were not ruled out. Although there are several case reports that suggested high doses of green tea extracts or catechin as a cause of liver damage, a relationship could not be easily established because the green tea supplements were often mixed with other ingredients, and some were not described as containing catechin.^10^ Furthermore, a case report, although suggestive, is hardly definitive because it is based on an implicit comparison with an ‘expected' or an usual experience.^11^ Without an appropriate control, it is not possible to determine whether the risk of liver damage differs among individuals exposed or not exposed to green tea supplements.

A randomized controlled trial (RCT) is the most rigorous method for estimating the impact of an intervention through explicit comparison with a concurrent control.^12^ So far, many RCTs of green tea intervention have been conducted and reported in the literature. In order to quantify the causal effects of existing interventions, systematic consideration based on RCTs of green tea intervention is required, regardless of the study's primary aims such as efficacy or safety. However, to the best of our knowledge, the systematic reviews published to date were only based on subjective assessments of case reports.^3, 9^ In order to more objectively examine the safety of green tea intake, we conducted a systematic review and collected data on liver-related adverse events reported in RCTs with a green tea intervention.

## Materials and methods

A systematic review was conducted with reference to the following relevant guidelines: Preferred Reporting Items for Systematic Reviews and Meta-Analyses^13^ and the Cochrane Handbook for Systematic Reviews of Interventions (particularly on adverse events).^14^

### Literature search

Articles on RCTs with oral intake of green tea were collected according to the following inclusion criteria: original articles; studies with human subjects; written in English; green tea alone orally administered in the intervention group; studies with concurrent ‘placebo' or ‘no-treatment' controls; and reports of adverse events. Green tea included not only beverages (liquids) but also green tea extracts used in tablets or powders. Studies on mixtures with ingredients containing anything other than components derived from green tea (catechin, theanine and caffeine) were excluded. Studies that included even small amounts of components derived from green tea (catechin, theanine and caffeine) in the placebo were excluded. If multiple articles were published on the same research, they were regarded as a single study, and the report on safety was taken as the main article, or the first article published was adopted. No scope was set for the search period.

The literature search was conducted with the following databases in December 2013: PubMed; Excerpta Medica Database (EMBASE); and Cochrane Central Register of Controlled Trials (CENTRAL). The search terms were (1) catechin, green tea extract, green tea polyphenol (GTP), green tea flavonoid, epigallocatechin gallate or EGCG and (2) RCT. The results of the search were listed for each database, and duplicate articles were excluded. Two independent reviewers then selected articles that were considered likely to meet the inclusion criteria from the title and abstract. The full-text versions of these selected articles were then independently screened by the same two reviewers to determine whether they met the inclusion criteria. Disagreements were resolved by discussion and consensus.

### Data abstraction and quality assessment

Data on adverse events related to the liver were extracted from the selected articles. In addition to liver disease (liver disorders, liver cancer, and so on), laboratory findings of abnormalities in liver function, for example, aspartate aminotransferase (AST), alanine transaminase (ALT), alkaline phosphatase (ALP), bilirubin, etc., were also included as events related to the liver. Adverse events were those that occurred during the intervention period. If an article described ‘no adverse events' or if only adverse events other than the liver were reported, adverse events related to the liver were regarded as no events.

To evaluate the results of adverse events, study methodology and reporting of adverse events must be taken into account. Thus, for each study, the following information was extracted: primary objective (efficacy or safety), type of design (parallel or crossover), masking (double-blind, single-blind or open-label), type of control group (placebo, water or no treatment), number of subjects, intake of green tea, intervention period and liver function monitoring. The number of subjects was the number of those who received at least one intervention. If the number of subjects was not noted, the number of subjects at the end of the study was used. Intake of green tea was regarded as intake per day. Intake was converted from one unit to another when necessary. Liver function monitoring included whether blood tests to evaluate liver function were performed.

Two reviewers independently extracted data and assessed the methodological quality of the included RCTs. Quality assessment was undertaken using the Cochrane Collaboration's tool for assessing risk of bias mainly related to randomization, allocation concealment, masking, incomplete outcome data and selective reporting.^15^ Risk of each bias was graded as low, high or unclear. Disagreements were resolved by discussion and consensus. If details of the adverse event were not described, confirmation was made by contacting the author of the article.

### Data analysis

The odds ratio (OR) was used to assess the risk of liver-related adverse events associated with green tea interventions. The OR and 95% confidence interval (CI) were calculated for each study, and an estimated summary OR for all studies was obtained using the Mantel–Haenszel method, assuming a fixed-effects meta-analysis model because of sparse event data with imbalanced study arms.^16^ In some studies included in the meta-analysis, there were no events reported in the control group. To avoid computational errors due to division by zero, all estimates were performed using a continuity correction. A ‘treatment arm' continuity correction, adding a factor of the reciprocal of the size of the opposite treatment arm, was applied because this correction has been shown to outperform the more usual constant continuity correction of 0.5 when the group imbalance is high.^16^ Other methods such as the Mantel–Haenszel method without a continuity correction and the Peto method were used to assess the robustness of the results. Statistical significance was assumed if the 95% CI did not overlap the null value (OR of 1).

Inconsistency across studies was evaluated with the *I*^2^ statistic, ranging from 0% (no observed heterogeneity) to 100%. An *I*^2^ statistical value of less than 25% was considered homogeneous.^17^

Studies in which there were no events in both arms were excluded from analyses. This was because such studies do not provide any indication of either the direction or magnitude of the relative intervention effect.^18^

Statistical analyses were conducted using StatsDirect version 3 (StatsDirect Ltd, Cheshire, UK).

## Results

### Literature search

**Figure 1:**
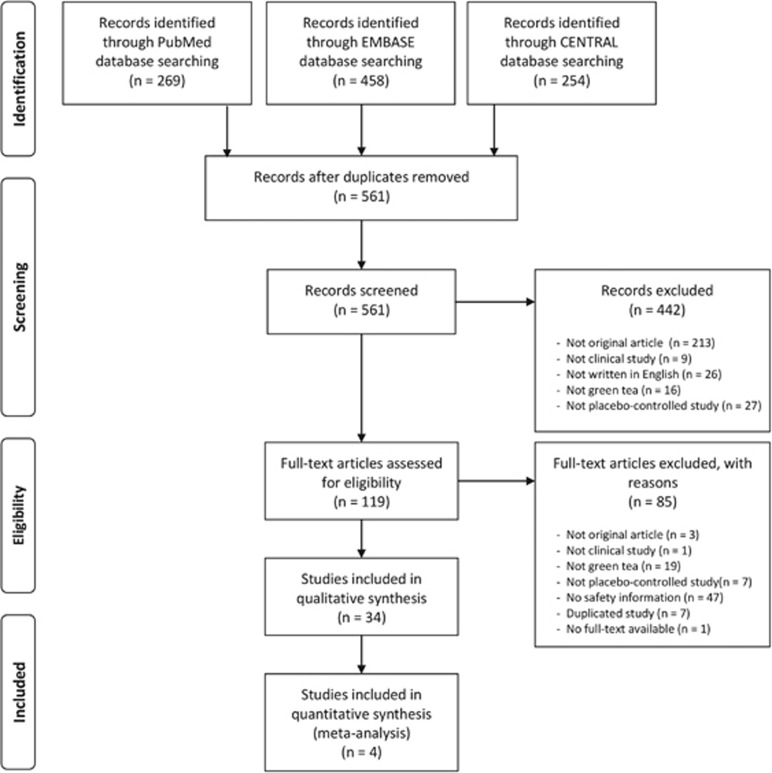
Flowchart of study selection.

The database search returned 269 PubMed articles, 458 EMBASE articles and 254 CENTRAL articles. Of these, 561 articles were extracted after duplicate articles were excluded. Articles that met the inclusion criteria were selected based on the title and abstract, and 119 proceeded to the full-text assessment, of which 34 were finally selected. The procedure for the literature search is shown as a flowchart in Figure 1.

### Study characteristics and quality assessment

An overview of the studies in the selected articles is shown in Table 1.^19, 20, 21, 22, 23, 24, 25, 26, 27, 28, 29, 30, 31, 32, 33, 34, 35, 36, 37, 38, 39, 40, 41, 42, 43, 44, 45, 46, 47, 48, 49, 50, 51, 52^ Among the 34 studies, 28 were studies with the primary objective of efficacy assessment and six were studies for safety assessment. The subjects were healthy (10 studies), obese (seven studies), cancer patients (five studies) or other (12 studies). The method of administration was repeated, except in one study with a single administration. The study durations were 2 years at the longest and 3 days at the shortest (excluding the one single-administration study).

As for masking, there were 24 double-blind studies, six single-blind studies and four open-label studies. With respect to design, there were 27 studies with parallel design, three studies with two-arm crossover with a washout period and four with a different crossover design. The types of control groups were 28 placebo, four water and two non-treatment. Of the 34 studies, 19 were randomized, double-blind, placebo-controlled studies, 13 of which used blood tests to assess liver function.

**Figure 2:**
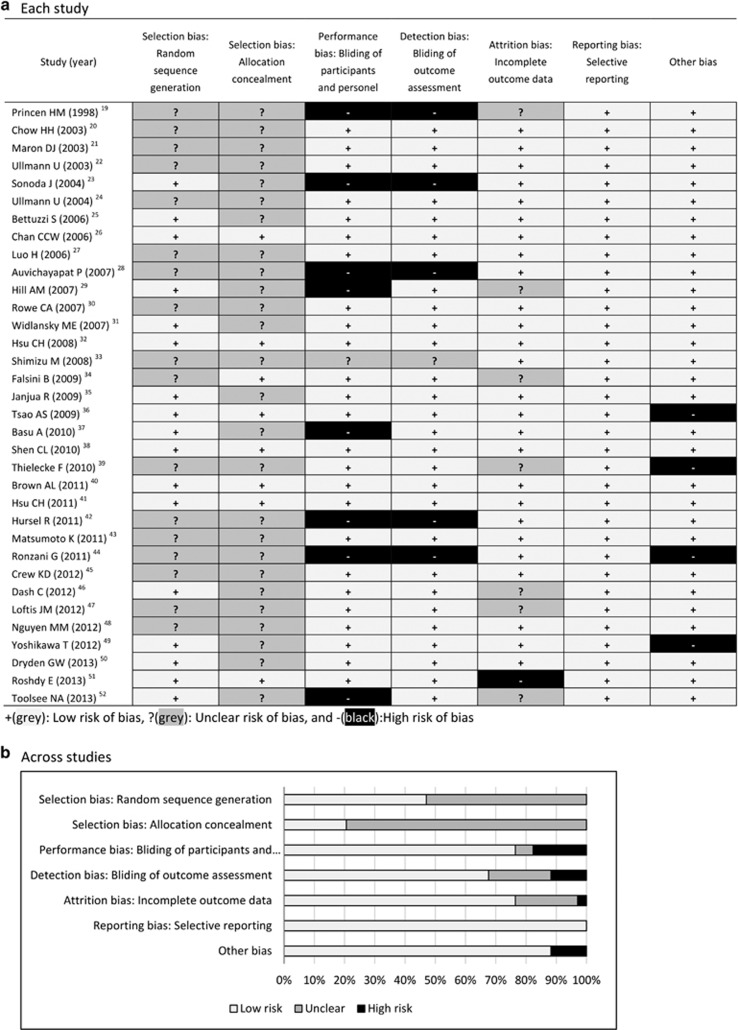
Risk of bias assessment.

Methodological quality was assessed for each included study, and results are summarized in Figure 2. Overall, most of the included RCTs were at low or unclear risk of bias for most items. Although all studies claimed randomization, many of them did not clearly describe the methods of randomization sequence generation and allocation concealment, making selection criteria unclear. There seemed to be no improvement in the methodological quality over time.

### Adverse events concerning liver damage

Liver-related adverse events were reported in 4 of the 34 studies (Table 2), but none of these were serious adverse events. Three of the four studies had the primary objective of safety assessment. All were double-blind studies where liver function was assessed by blood tests.

When data for the selected studies were simply integrated, reported adverse events related to the liver were seven (eight events) in 1405 subjects (0.5%) in green tea groups and one (one event) in 1200 subjects (0.1%) in control groups. On the basis of green tea intake, there was one event at 500 mg/day, three events at 800 mg/day, one event at 1200 mg/day and three events at 1600 mg/day.

**Figure 3:**
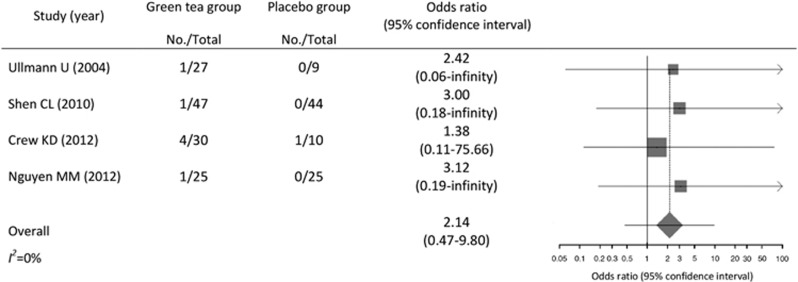
Meta-analysis of reported liver-related adverse events. Note: the size of the square is proportional to the weight of the study.

The summary OR for liver-related adverse events in subjects who received green tea intervention versus placebo was 2.1 (95% CI, 0.5–9.8; Figure 3). The estimates remained similar when applying the Mantel–Haenszel method without a continuity correction (OR, 3.3; 95% CI, 0.4–26.8) and the Peto method (OR, 2.6; 95% CI, 0.5–12.9). All results were not statistically significant. No heterogeneity was observed across studies (*I*^*2*^=0%).

The liver-related adverse events reported are summarized below by study.

Ullmann *et al.*^24^ conducted a double-blind, randomized, placebo-controlled trial in healthy males to examine the safety, tolerability and pharmacokinetics of EGCG. The 36 subjects were randomly allocated into three groups of 12 (EGCG 200, 400 and 800 mg) and were treated once a day for 10 days. Three subjects in each group received the placebo. Safety was assessed from blood tests (including liver function assessment), vital signs and physical findings before and 10 days after the start of administration, as well as from the subjects' self-reports. Slightly elevated ALT was seen at the end of study in one subject in the 800-mg group; however, it returned to the normal range within 14 days after the study. This event was judged to have no relation to EGCG administration. No dose–response relationship was observed.

Shen *et al.*^38^ conducted a randomized, placebo-controlled trial in postmenopausal women to assess the effects of both GTPs and exercise on osteopenia. With the primary objective of safety, the 171 subjects were randomly allocated into four groups (GTP, placebo, GTP+exercise and placebo+exercise) and were administered GTP 500 mg/day for 24 weeks. The use of either GTP or placebo was double blind. Safety was assessed from the subjects' self-reports and blood tests, including liver function assessment, at baseline and every 4 weeks. In the 85 subjects who received GTP (47 subjects in the GTP group and 38 subjects in the GTP+exercise group), elevated AST and ALT, which were possibly effects of concomitant medication for cold symptoms, were observed in one subject in the GTP group. This subject had taken ibuprofen (400 mg/day for 9 days) as cold medication, Lipitor (20 mg/day) to treat hyperlipidemia and metoprolol (25 mg/day) to treat hypertension. AST and ALT returned to the normal range when the subject stopped taking ibuprofen. This event was judged to have no relation to GTP administration.

Crew *et al.*^45^ conducted a phase 1b double-blind, randomized, placebo-controlled, dose-escalation trial of green tea extract (Polyphenon E, Mitsui Norin, Japan) in women with a history of breast cancer, and the maximum tolerated dose for 6-month administration was determined. The 40 subjects were allocated with 10 subjects in the placebo group and 16, 11 and 3 subjects in the green tea extract 400-, 600- and 800-mg groups, respectively. Administration was twice a day (total of 800-, 1200- or 1600-mg EGCG daily) for 6 months. Safety was assessed by monitoring adverse events and laboratory values at baseline, every 2 weeks for 1 month after the start of administration and once a month thereafter. In the EGCG 800-mg group, one case of mildly elevated ALP was reported 17 days after the start of administration. In the EGCG 1200-mg group, one case of mildly elevated ALP was reported 30 days after the start of administration. In the 1600-mg/day group, one case of severely elevated ALT was reported 91 days after the start of administration, and administration was discontinued. In addition, one case of mild transaminitis was reported 91 and 119 days after the start of administration. In the placebo group, one case of mildly elevated bilirubin was reported.

Nguyen *et al.*^48^ conducted a double-blind, randomized, placebo-controlled trial to determine the bioavailability of green tea extract (Polyphenon E) in patients with prostate cancer before radical prostatectomy. The 50 subjects were randomly allocated, with 25 in the green tea extract group and 25 in the placebo group. Administration was once daily (EGCG 800 mg) for 3–6 weeks. Safety was assessed from the subjects' self-reports and blood tests (including liver function assessment), at the start and end of administration. In the results, mildly elevated ALT was observed in one subject in the green tea extract group.

## Discussion

In this review, liver-related safety of green tea intervention was assessed through a systematic review of published RCTs, allowing for a direct comparison with controls. Most of the RCTs selected reported no liver-related adverse events in either intervention or control groups. The few events reported in intervention groups were elevations of liver enzymes such as ALT or ALP. No serious adverse events were reported; most were mild, but one severe adverse event was reported, leading to the discontinuation of intervention. None of the events were judged to have a definite causal relationship to green tea intake.

Although meta-analyses were conducted using methods for sparse event data, the results were inconclusive because of the lack of studies with events. The summary ORs implied a possible risk of liver damage compared with controls but remained uncertain with wide CIs overlapping the null value. Few studies with few events were likely to cause imprecision of estimates; however, beyond methodology, instability seems inherent in estimation involving very few events. Despite possible risk, the majority of the studies selected reported no liver-related adverse events even in the intervention groups. Further research based on a well-designed RCT, with adequate sample size, is warranted to provide a more precise estimation of green tea intervention effects.

In the present review, almost all of the liver-related adverse events were derived from safety studies, whereas most of the studies selected were efficacy studies; events were noted in one of the 28 efficacy studies and in three of the six safety studies. Two of these three safety studies were dose-escalation studies to evaluate the safety and tolerability of intervention, where a relatively high dose of intervention was applied, and adverse events were more likely to be encountered compared with other types of studies. Reporting of adverse events tends to depend on the purpose of the study;^53, 54^ therefore, in this review, the study objective was not added to the inclusion criteria in order to collect as many results of green tea intervention as possible. Safety often receives less attention in efficacy trials;^55^ hence, it is unlikely that the occurrence of adverse events would become a direct obstacle to publication (that is, publication bias). Although most of the efficacy studies selected did not report liver-related adverse events, the results did not seem to be particularly distorted.

Liver damage often leads to liver cancer; however, there were no reports of liver cancer in the studies selected. To date, many epidemiological studies have been conducted, expecting anticancer effects from green tea or catechin. Of several recent prospective cohort studies to evaluate the effects of green tea consumption on liver cancer,^56, 57, 58, 59^ only the study conducted by Ui *et al.*^58^ showed a statistically significant decrease in cancer risk. A systematic review of the cancer-preventing effects of green tea extracts conducted by the Cochrane Collaboration also did not show any clear preventive effects on liver cancer.^60^ Similar results were obtained in systematic reviews of the preventive effects of green tea beverages on liver cancer.^61, 62^ Although there is still no clear evidence of the preventive effects of green tea consumption, no studies have reported an increased risk of developing liver cancer. As these epidemiological studies assessed the prevention of liver cancer (that is, efficacy), consideration of the aspects of harm was limited; however, the fact that no liver cancer reports were found in this review seems reasonable.

This study had several limitations that should be noted. First, the present review included only English language studies, which may have caused reports in other languages to be missed. Thus, to identify as many relevant studies as possible, a broad search was undertaken using three large databases with a wide timespan from inception to December 2013. Second, a majority of the studies were at unclear risk of selection bias. Details of randomization and allocation concealment were not adequately clarified, although the present review only included the studies claiming randomization. Methodology details should be included in future studies. Third, most studies were relatively short (median: 12 weeks), possibly resulting in few liver-related adverse events. Usually, liver injury induced by drugs or herbal medicines occurs within 6 months after initiation; hence, routine monitoring of liver function is offered in the first 6 months of treatment.^63, 64^ However, case reports in the literature have shown that the treatment period by the time such liver injury occurred varied between cases.^3, 9^ It seems that assessment based on a broad range of treatment periods is more important than the duration of treatment, which was achieved in the present review. Finally, the literature search for this review identified only studies on green tea extract products. However, traditional green tea infusions or other beverage preparations are considered to be safe because of their widespread and long history of use.^3, 9^ Despite these limitations, compared with the study populations in single RCTs, the broad range of study characteristics in this review strengthens the generalizability of our findings.

In conclusion, the results of the present review suggest that liver-related adverse events after intake of green tea extracts are expected to be rare. However, consumers should always be provided with updated safety information by manufacturers, and care must be taken to follow product recommendations in order to minimize any potential risks.

## Figures and Tables

**Table 1:** Characteristics of selected studies

*Study (reference)*	*Study aim*	*Participants*	*Intervention group: intake details (total daily dose)*^a^	n	*Control group*	n	*Duration*	*Study design*	*Masking*	*Blood test for liver function*
Princen *et al.*^19^	Efficacy	Healthy volunteers	Green tea beverage: six cups (150 ml each) daily (851.7 mg catechins)	15^b^	Water	15^b^	4 Weeks	Parallel	Single	—
			Green tea polyphenol isolate CAPs: 4 CAPs 6 times daily (2488.7 mg catechins)	13^b^						
Chow *et al.*^20^	Safety	Healthy volunteers	EGCG CAPs: four CAPs daily (800 mg EGCG)	8	Placebo	8	4 Weeks	Parallel	Single	Yes
			EGCG CAPs: 2 CAPs twice daily (800 mg EGCG)	8						
			Poly E CAPs: 4 CAPs daily (800 mg EGCG)	8						
			Poly E CAPs: 2 CAPs twice daily (800 mg EGCG)	8						
Maron *et al.*^21^	Efficacy	Adults with mild to moderate hypercholesterolemia	GTE CAPs: one CAP daily (150 mg catechins)	120	Placebo	120	12 Weeks	Parallel	Double	Yes
Ullmann *et al.*^22^	Safety	Healthy men	EGCG CAPs: one CAP daily (50 mg EGCG)	8	Placebo	2	Single dose	Parallel	Double	—
			EGCG CAPs: two CAPs daily (100 mg EGCG)	8	Placebo	2				
			EGCG CAPs: four CAPs daily (200 mg EGCG)	8	Placebo	2				
			EGCG CAPs: eight CAPs daily (400 mg EGCG)	8	Placebo	2				
			EGCG CAPs: 16 CAPs daily (800 mg EGCG)	8	Placebo	2				
			EGCG CAPs: 32 CAPs daily (1600 mg EGCG)	8	Placebo	2				
Sonoda *et al.*^23^	Efficacy	Asymptomatic HTLV-1 carriers	GTE CAPs: nine CAPs daily (245.7 mg EGCG)	47	No treatment	48	5 Months	Parallel	Open	—
Ullmann *et al.*^24^	Safety	Healthy men	EGCG CAPs: four CAPs daily (200 mg EGCG)	9	Placebo	3	10 Days	Parallel	Double	Yes
			EGCG CAPs: eight CAPs daily (400 mg EGCG)	9	Placebo	3				
			EGCG CAPs: 16 CAPs daily (800 mg EGCG)	9	Placebo	3				
Bettuzzi *et al.*^25^	Efficacy	High-grade prostate intraepithelial neoplasia	Green tea catechin CAPs: three CAPs daily (600 mg catechins)	30	Placebo	30	1 Year	Parallel	Double	—
Chan *et al.*^26^	Efficacy	Women with polycystic ovary syndrome	Green tea CAPs: sic CAPs daily (540 mg EGCG)	18	Placebo	16	3 Months	Parallel	Double	—
Luo *et al.*^27^	Efficacy	Patients at high-risk of liver cancer	GTP CAPs: four CAPs daily (500 mg GTPs)	42	Placebo	41	3 Months	Parallel	Double	Yes
			GTP CAPs: four CAPs daily (1000 mg GTPs)	41						
Auvichayapat *et al.*^28^	Efficacy	Obese	Green tea CAPs: three CAPs daily (750 mg GTE)	30	Placebo	30	12 Weeks	Parallel	Single	—
Hill *et al.*^29^	Efficacy	Overweight post-menopausal women	EGCG CAPs: two CAPs daily (300 mg EGCG)	19^b^	Placebo	19^b^	12 Weeks	Parallel	Single	Yes
Rowe *et al.*^30^	Efficacy	Healthy volunteers	Green tea CAPs: one CAP twice daily	55	Placebo	53	12 Weeks	Parallel	Double	—
Widlansky *et al.*^31^	Efficacy	Patients with CAD	EGCG CAPs: one CAP twice daily (300 mg EGCG)	54	Placebo	52	2 Weeks	Crossover (two-arm, 1-week washout)	Double	Yes
Hsu *et al.*^32^	Efficacy	Obese women	GTE CAPs: three CAPs daily (1200 mg GTEs)	50	Placebo	50	12 Weeks	Parallel	Double	Yes
Shimizu *et al.*^33^	Efficacy	Healthy volunteers	GTE TABs: three TABs daily (1500 mg GTEs)	68	Water	65	12 Months	Parallel	Open	—
Falsini *et al.*^34^	Efficacy	Ocular hypertension and glaucoma	EGCG oral treatment: 200 mg EGCG oral treatment daily	36^b^	Placebo	36^b^	3 Months	Crossover (two-arm, no washout)	Double	—
Janjua *et al.*^35^	Efficacy	Healthy women	Green tea CAPs: one CAP twice daily (350 mg catechins)	29	Placebo	27	2 Years	Parallel	Double	—
Tsao *et al.*^36^	Efficacy	High-risk oral premalignant lesions	GTE CAPs: 500 mg/m^2^ GTE daily	11	Placebo	11	12 Weeks	Parallel	Double	Yes
			GTE CAPs: 750 mg/m^2^ GTE daily	9						
			GTE CAPs: 1000 mg/m^2^ GTE daily	10						
Basu *et al.*^37^	Efficacy	Obese and metabolic syndrome	Green tea beverage: four cups daily (928 mg catechins)	13^b^	Water	12^b^	8 Weeks	Parallel	Single	Yes
			GTE CAPs: two CAPs daily (870 mg catechins)	10^b^						
Shen *et al.*^38^	Efficacy	Postmenopausal women	GTP CAPs: two CAPs daily (500 mg GTPs)	47	Placebo	44	24 Weeks	Parallel	Double	Yes
Thielecke *et al.*^39^	Efficacy	Overweight men	EGCG CAPs: one CAP twice daily (300 mg EGCG)	10	Placebo	10	3 Days	Crossover (five-arm, ⩾7- day washout)	Double	—
			EGCG CAPs: one CAP twice daily (600 mg EGCG)	10						
Brown *et al.*^40^	Efficacy	Overweight men	Decaffeinated GTE CAPs: one CAP twice daily (1060 mg GTEs)	69	Placebo	72	6 Weeks	Crossover (two-arm, ⩾2- week washout)	Double	Yes
Hsu *et al.*^41^	Efficacy	Obese type 2 DM patients	Decaffeinated GTE CAPs: one CAP three times daily (1500 mg GTEs)	40	Placebo	40	16 Weeks	Parallel	Double	Yes
Hursel *et al.*^42^	Efficacy	Healthy volunteers	Green tea CAPs: three CAPs daily (507 mg catechins)	18	Placebo	18	1 Week	Crossover (six-arm, no washout)	Single	—
Matsumoto *et al.*^43^	Efficacy	Healthy volunteers	Green tea CAPs: six CAPs daily (378 mg catechins)	97	Placebo	99	5 Months	Parallel	Double	—
Ronzani *et al.*^44^	Efficacy	Patients with breast cancer	GTE TABs: two TABs twice daily (800 mg catechins)	46	No treatment	46	3 Weeks	Crossover (two-arm, 1-day washout)	Open	—
Crew *et al.*^45^	Safety	Women with hormone receptor-negative breast cancer	Poly E CAPs: two CAPs twice daily (800 mg EGCG)	13^b^	Placebo	8^b^	6 Months	Parallel	Double	Yes
			Poly E CAPs: three CAPs twice daily (1200 mg EGCG)	6^b^						
			Poly E CAPs: four CAPs twice daily (1600 mg EGCG)	1^b^						
Dash *et al.*^46^	Efficacy	Smoker and non-smoker	Green tea beverage: five packets daily (640 mg catechins)	45	Placebo	48	4 Weeks	Crossover (4-arm, 2- week washout)	Double	—
Loftis *et al.*^47^	Efficacy	Schizophrenia and bipolar disorder	GTE CAPs: two CAPs twice daily (600 mg GTE)	16	Placebo	15	8 Weeks	Parallel	Double	Yes
Nguyen *et al.*^48^	Efficacy	Prostate cancer	Poly E CAPs: four CAPs daily (800 mg EGCG)	25	Placebo	25	3–6 Weeks	Parallel	Double	Yes
Yoshikawa *et al.*^49^	Efficacy	Healthy volunteers	GTE CAPs: three CAPs three times daily (1069.4 mg catechins)	20	Placebo	20	1 Weeks	Parallel	Double	Yes
Dryden *et al.*^50^	Efficacy	Patients with mild to moderate ulcerative colitis	Poly E CAPs: one CAP twice daily (400 mg EGCG)	5^b^	Placebo	1^b^	56 Days	Parallel	Double	Yes
			Poly E CAPs: two CAPs twice daily (800 mg EGCG)	8^b^	Placebo	2^b^				
Roshdy *et al.*^51^	Efficacy	Women with uterine fibroids	GTE CAPs: two CAPs daily (360 mg EGCG)	22	Placebo	17	4 Months	Parallel	Double	Yes
Toolsee *et al.*^52^	Efficacy	Prediabetic mauritians	Green tea beverage: one cup (200 ml) three times daily (1519.7 mg green tea catechins)	77	Water	78	14 Weeks	Parallel	Open	Yes

Abbreviations: CAD, coronary artery disease; CAP, capsule; DM, diabetes mellitus; EGCG, epigallocatechin gallate; GTE, green tea extract; GTP, green tea polyphenol; HTLV-1, human T-cell lymphotropic virus type 1; Poly E, polyphenon E; TAB, tablet.

a Intake details shown as explained in the reference, and total daily dose if available in the reference.

b Number of subjects who completed the study.

**Table 2:** Summary of reported liver-related adverse events

*Study (reference)*	*Group*	*Total daily dose*^a^	n	*Adverse event*	*Remarks*
Ullmann *et al.*^24^	Green tea	800 mg EGCG	1	Elevated ALT level	
Shen *et al.*^38^	Green tea	500 mg GTPs	1	Elevated AST and ALT levels	Possibly due to concomitant medications
Crew *et al.*^45^	Control	—	1	Hyperbilirubinemia	Grade 1
	Green tea	800 mg EGCG	1	High alkaline phosphatase	Grade 1
	Green tea	1200 mg EGCG	1	High alkaline phosphatase	Grade 1
	Green tea	1600 mg EGCG	1	Transaminitis	Two events, both were Grade 1
	Green tea	1600 mg EGCG	1	Elevated ALT level	Grade 3
Nguyen *et al.*^48^	Green tea	800 mg EGCG	1	Elevated ALT level	Grade 1

Abbreviations: ALT, alanine transaminase; AST, aminotransferase; EGCG, epigallocatechin gallate; GTP, green tea polyphenol.

Note: Grades were judged by the National Cancer Institute Common Terminology Criteria for Adverse Events (Version 3.0): Grade 1 for mild and Grade 3 for Severe.

a Total daily dose expressed as stated in the reference.
